# From the First to the Second Wave of COVID-19: Anxiety, De-Pressive, and Post-Traumatic Stress Symptoms in the Italian Population

**DOI:** 10.3390/ijerph19031239

**Published:** 2022-01-22

**Authors:** Agata Benfante, Valentina Tesio, Marialaura Di Tella, Annunziata Romeo, Lorys Castelli

**Affiliations:** Department of Psychology, University of Turin, 10124 Torino, Italy; agata.benfante@unito.it (A.B.); marialaura.ditella@unito.it (M.D.T.); annunziata.romeo@unito.it (A.R.); lorys.castelli@unito.it (L.C.)

**Keywords:** COVID-19, anxiety symptoms, depressive symptoms, post-traumatic stress symptoms, longitudinal design, Italian population

## Abstract

In the early stages of the COVID-19 outbreak, high rates of clinically relevant anxiety, depression, and post-traumatic stress symptoms (PTSS) have been reported in the Italian population. The persistence of the pandemic and related restrictive measures highlight the need for a reassessment of psychopathological symptoms. The present longitudinal study consisted of two evaluations conducted during the two waves of infection. Participants were asked to complete the State-Trait Anxiety Inventory-Form Y1 (STAI Y1), the Beck Depression Inventory (BDI-II), and the PTSD Checklist for DSM-5 (PCL-5). There were no significant differences in depressive symptoms and PTSS scores reported by participants between T0 and T1, with single-case analysis revealing that in 71% and 69% of the participants, depressive symptoms and PTSS symptoms, respectively, remained stable during this period. On the contrary, mean scores comparison showed a significant decrease in anxiety levels, with 19% of participants in whom anxiety symptoms improved at single-case analysis. Taken together, these results suggest that depressive symptoms and PTSS not only occurred in a high percentage of participants but also tended to remain stable over time, thus warranting the importance of large-scale psychological screening and interventions to prevent the chronicization of these symptoms and their evolution to psychopathological disorders.

## 1. Introduction

In December 2019, the first SARS-CoV-2 infections were reported in China. The world was plagued by the COVID-19 outbreak in a matter of months [1]. The population had to adapt to drastic changes in routine life and live with constant fear of contagion. It was immediately evident that an event of this magnitude would have a negative impact on the mental health of the general population [2,3] and certain sub-populations, such as healthcare workers, who were directly affected by the consequences of the disease [4,5]. 

Since 9 March 2020, several restrictive measures have been introduced in Italy to contain the infections, defining different scenarios based on infection data trends [6]. In particular, two so-called “waves of infection” emerged, with an increasing number of cases. Specific to Italy, the first wave began in March 2020, followed by a decline in cases in early summer, and the second wave began in November 2020.

A few weeks following the onset of the March 2020 lockdown, a study investigated the psychological impact of COVID-19 on the Italian population. The results showed that at the early stage of the pandemic, 69%, 31%, and 20% of the 1321 participants in the study, respectively, reported scores higher than the cut-off for measures of anxiety, depression, and post-traumatic stress symptoms (PTSS), suggesting the presence of clinically relevant symptoms [7]. Similarly, other studies have found high levels of psychological distress in this population [8,9].

Psychological symptoms, caused by a traumatic event, might become chronic over time and, eventually, they might lead to psychopathological disorders [10,11,12]. However, a segment of the population with minor psychological symptoms might remain unrecognized, depending on the methodologies and instruments used in studies. Individuals who did not obtain a score indicating the presence of clinically relevant symptoms might still experience some minor mental health symptoms [13]. The symptoms could be present at a sub-clinical level in these individuals and have negative implications for their psychological well-being.

The findings regarding the first wave of the contagion, along with the persistence of the pandemic, highlighted the need for a new investigation of psychopathological symptoms. 

Thus, the present longitudinal study aimed to assess the prevalence of anxiety/depressive symptoms and PTSS during the two waves of COVID-19 contagion to monitor the evolution of those psychopathological symptoms over time. 

## 2. Materials and Methods

Data were collected using an anonymous online survey during the first wave of COVID-19 (T0) from 19 March 2020 to 5 April 2020, with a snowball sampling (for the results, see [7]). At the end of the questionnaire, participants were asked about the possibility of participating in the follow-up of the study, asking them to provide their email addresses in order to get the link to the second phase. For the second wave (T1) from 4 December 2020 to 10 January 2021, the survey was completed by 332 participants, i.e., 34% of the 979 individuals who agreed to be contacted for this second evaluation. Participants were asked to provide sociodemographic and clinical information and complete the following three self-report questionnaires: (1) State-Trait Anxiety Inventory-Form Y1 to evaluate the presence of anxiety symptoms; (2) Beck Depression Inventory (BDI-II) to assess the levels of depressive symptoms; and (3) PTSD Checklist for DSM-5 (PCL-5) to examine post-traumatic stress symptoms (PTSS). 

First, descriptive analysis was used to investigate the psychological impact of the first and second waves of COVID-19 on the participants. Second, paired t-tests (for continuous variables) and exact McNemar’s tests (for dichotomous variables) were performed to compare the levels of anxiety/depressive symptoms and PTSS at T0 and T1. Third, single case change scores were calculated by subtracting the score at T1 from the score at T0 [14]. Z-scores were then computed for these newly created variables to identify participants who reported a significant increase/decrease in psychopathological symptoms (i.e., participants who scored one standard deviation above or below the mean, respectively) or who were stable at T1 compared to T0.

Finally, participants who reported a significant increase or decrease in those symptoms were compared by means of independent t-tests (for continuous variables) and chi-squared tests (for dichotomous variables).

This study was approved by the University of Turin’s ethics committee (protocol n. 488755) and was conducted in accordance with the Declaration of Helsinki. All participants provided informed consent prior to participation.

## 3. Results

Sociodemographic data of the total sample are shown in Table 1. Participants were between 18 and 82 years old, with an average age of about 35 years.

Psychological data of the total sample are shown in Table 2. There were no significant differences in depressive symptoms and PTSS between T0 and T1 (BDI-II: t(331) = −0.767, *p* = 0.444, d = 0.06; PCL-5: t(331) = −1.384, *p* = 0.167, d = 0.10), but there was a significant difference in anxiety symptoms between T0 and T1 (STAI Y1: t(331) = 4.198, *p* < 0.001, d = 0.33), with participants reporting lower anxiety scores at T1 when compared to T0. Consistent with these results, an exact McNemar’s test revealed a statistically significant difference in the proportion of individuals who scored above the cut-off point for the STAI Y1 at T0 versus T1 (*p* < 0.001), while no significant differences were detected for the BDI-II (*p* = 0.518) and the PCL-5 (*p* = 0.431).

A further single-case analysis was conducted to investigate change scores (score at T1 minus score at T0 transformed in Z-scores) for the BDI-II, PCL-5, and STAI Y1 to identify participants who showed a significant change (less/more symptoms or stable) between T0 and T1 (see Table 3 for results). 

Comparisons of sociodemographic (age, gender, education, marital status, and parental status) and clinical (having/not having a medical condition) variables between participants who reported a significant increase and those who reported a decrease in psychopathological symptoms revealed statistically significant differences for depressive symptoms based on gender (*χ*^2^(1) = 4.457, *p* = 0.035) and marital status (*χ*^2^(1) = 4.801, *p* = 0.028). In particular, being female and in a relationship was found to be significantly associated with a worsening of depressive symptoms. In contrast, no statistically significant differences in sociodemographic or clinical variables were detected between participants with an increase or decrease in PTSS and anxiety scores (all *p* > 0.05). 

## 4. Discussion

The main aim of the present study was to shed light on changes in the levels of anxiety/depressive symptoms and PTSS in the Italian population during the first and second waves of the COVID-19 outbreak. Comparisons of data from the two outbreak waves revealed no significant differences in depressive symptoms or PTSS but a significant decrease in anxiety levels. These results are consistent with those of an earlier Italian study, which found no differences in clinically relevant depressive symptoms and a significant reduction in anxiety [15]. 

According to one possible explanation for the findings, anxiety may be a normal initial response to a highly stressful event and an unknown threat. Indeed, uncontrolled fears of infection and pervasive anxiety are among the most relevant psychological reactions to COVID-19 [16]. The virus is gradually becoming more understood, and it is plausible that anxiety levels can be reduced. Conversely, results of the present study suggest that during the new wave of COVID-19 infection and the new constraints, individuals experienced the same levels of depressive symptoms and PTSS as during the first wave, thus outlining a chronicization of these symptoms. 

While this evidence supports some studies (e.g., [16]), it contradicts others (e.g., [17]). There could be multiple explanations for the discrepant findings.

Some longitudinal studies, for example, have made subsequent evaluations during the first lockdown [18,19,20], suggesting a worsening of mental health in the general population from the early phases to the peak of the transmission of the contagion. Other studies have focused on the change of symptoms between the first and second phases of the pandemic [17,21]. A study conducted by Li et al. [17] on Chinese students revealed a decrease in acute stress symptoms, while anxiety levels and depressive symptoms increased from the COVID-19 outbreak to the COVID-19 remission stage. Conversely, González-Sanguino et al. [21] found a significant increase in depressive symptoms over time in three evaluations at the onset, at the most difficult moment, and at de-escalation of the first Spanish quarantine, a downward trend with a significant difference between the first and the third evaluations in PTSS and no significant changes in anxiety scores, which remained stable throughout the lockdown [21].

Results from the single-case analysis revealed that, consistent with group analysis, the majority of the participants showed no significant changes between T0 and T1. Regarding anxiety, a slightly high percentage of participants, about 19%, showed a significant improvement in anxiety symptoms, while about 15% of participants showed significant worsening of depressive or PTSS symptoms. Participants who reported an increase in depressive symptoms were more likely to be female and in a relationship than those who reported a significant decrease in psychopathological symptoms. 

This last piece of evidence highlighted that being female [18,21] and in a relationship [22] is associated with poorer mental health in response to the pandemic, as well as a higher risk of depressive symptoms worsening over time.

## 5. Conclusions

In conclusion, the present study confirmed the high prevalence of depressive symptoms and PTSS during the second wave. Clinically relevant depressive symptoms and PTSS did not appear to decrease over time, but rather remained high (point prevalence) and stable. These results are even more worrying when compared with the prevalence of psychiatric disorders in the Italian population outside the pandemic period. The European Study on the Epidemiology of Mental Disorders, part of the WHO World Mental Health Survey Initiative (ESEMeD-WMH), estimated the 12-month prevalence of any mood disorders at 3.5%, any anxiety disorders at 5.1%, and post-traumatic stress disorder at 0.8% in the adult Italian population [23]. The persistence of high levels of depressive symptoms and PTSS in the second wave, combined with a low percentage of individuals in whom these symptoms spontaneously resolve, underlines the high risk of chronicity, which can lead to the development of full-blown psychiatric syndromes.

From a public health perspective, these data provide a double clue. On the one hand, they indicate that during special events, such as the COVID-19 pandemic, it is essential to intervene immediately with preventive measures to protect mental health in the most vulnerable population. On the other hand, these results point to the current need to carry out interventions aimed at verifying the possible chronicity of these symptoms in the Italian population, in order to intervene with more appropriate psychological treatments.

This study has some limitations that should be considered. First, the second evaluation was conducted on a relatively small sample size, with a moderate response rate among participants who agreed to follow up. This may also have affected the single-case analysis, as the calculation of z-scores is closely linked to the sample. For these reasons, our findings should be considered with caution. Second, the sample had a higher proportion of females and well-educated participants.

Despite these limitations, the present study represents, to the best of our knowledge, the first attempt at investigating the trajectory of psychopathological symptoms in the Italian population during the COVID-19 outbreak. The findings show that the COVID-19 pandemic has had greater negative impact on women than on men, with the former showing a worsening of depressive symptoms over time. Therefore, psychological interventions are necessary to prevent the chronicity and exacerbation of mental health symptoms, especially for at-risk groups.

## Figures and Tables

**Table 1:** Sociodemographic data of the total sample (N = 332).

	Mean (SD)	N (%)
Age (years)	35.30 (13.97)	
Female		253 (76.2)
Male		79 (23.8)
Primary/Secondary/High school diploma		90 (27.1)
B.Sc. or M.Sc. Degree/Postgraduate qualification		242 (72.9)
In a relationship		128 (38.6)
Not in a relationship		204 (61.4)
*Children*		
Yes		102 (30.7)
No		230 (69.3)
*Medical Condition*		
Yes		66 (19.9)
No		266 (80.1)

SD = Standard Deviation.

**Table 2:** Psychological data of the total sample (N = 332). Mean (SD) and number of patients scoring above the cut-off have been reported as cases, at both T0 and T1.

Psychological Evaluation	Mean (SD)		N (%) of Cases ^1^	
	T0	T1	T0	T1
STAI Y1	48.44 (12.96)	44.15 (13.04) ^2^	225 (67.8)	178 (53.6) ^2^
BDI-II	10.58 (8.26)	11.09 (9.01)	108 (32.5)	117 (35.2)
PCL-5	18.94 (14.96)	20.59 (15.88)	62 (18.7)	71 (21.4)

SD = Standard Deviation; STAI Y1 = State-Trait Anxiety Inventory Form Y1; BDI-II = Beck Depression Inventory; PCL-5 = PTSD Checklist for DSM-5. ^1^ Participants who scored above the BDI-II cut-off point (≥14), the STAI Y1 cut-off point (≥41), and the PCL-5 cut-off point (≥33). ^2^ Statistically significant difference compared to T0.

**Table 3:** Number (percentage) of participants who reported a significant change in psychopathological symptoms or remained stable between T0 and T1 (N = 332).

Symptoms T0-T1	STAI-Y1	BDI-II	PCL-5
Stable	215 (64.8)	234 (70.5)	228 (68.7)
Worsened	53 (16.0)	47 (14.2)	49 (14.8)
Improved	64 (19.3)	51 (15.4)	55 (16.6)

BDI-II = Beck Depression Inventory; STAI Y1 = State-Trait Anxiety Inventory Form Y1; PCL-5 = PTSD Checklist for DSM-5.

## Data Availability

All relevant data are within the manuscript. The dataset used and/or analyzed during the current study is available from the corresponding author on reasonable request.
